# Supplementary material for: How Salt Solvation Slows Water Dynamics While Blue-Shifting Its Dielectric Spectrum
Source: J Phys Chem Lett. 2025 Jul 28;16(31):7915–20. doi: 10.1021/acs.jpclett.5c01401 (PMC12337140; doi:10.1021/acs.jpclett.5c01401)
Supplement: Supplementary file 1 [file jz5c01401_si_001.pdf]

How Salt Solvation Slows Water Dynamics While Blue-Shifting Its Dielectric Spectrum

SUPPORTING INFORMATION

Florian Pabst*
SISSA – Scuola Internazionale Superiore di Studi Avanzati, 34136 Trieste (Italy, EU)

Stefano Baroni
SISSA – Scuola Internazionale Superiore di Studi Avanzati, 34136 Trieste (Italy, EU) and CNR-IOM, Istituto dell'Officina dei Materiali, SISSA unit, 34136 Trieste (Italy, EU)

I. NEURAL NETWORK POTENTIAL

The Neural-Network Potential (NNP) used in this work is trained via a recently proposed "on-the-fly" learning procedure called Deep Potential Generator (DP-GEN) [S1]. The workflow consists of three steps which are repeated until convergence of the learning procedure is achieved. The first step is the training of four NNPs, differing only by the initialization seed, which is done in the very first iteration on data taken from short ab-initio simulations on concentrations spanning from pure water to the solubility limit of $Ca(ClO_4)_2$. These NNPs are then used in the second step to run short simulations in the NVT or NPT ensemble with temperatures up to 800 K and pressures up to 10 kbar. The difference in the forces predicted by the four NNPs is used as a criterion for selecting a snapshot for inclusion in the next training iteration. Forces and energies are calculated for the selected snapshots in the third step using density functional theory (DFT) with Quantum ESPRESSO™ [S2]. The RPBE functional and D3(BJ) dispersion correction is used for these calculations [S3, S4], as well as optimized norm-conserving Vanderbilt pseudo-potentials [S5] and a kinetic energy cutoff of 100 Ry. The training of the final NNP is done on 13960 configurations for 2 million steps. The cutoff radius was set to 6 Å and the size of the embedding and fitting nets is (25, 50, 100) and (240, 240, 240), respectively. After the training, the NNP was compressed to increase the speed of the simulations [S6]. Fig. S1 shows predicted atomic forces of the NNP for 100 configurations, not contained in the training set, versus DFT values. The average error on the forces is 38 meVÅ^{-1}, which is well below the threshold of 50 meVÅ^{-1} commonly considered as accurate for a DeepMD potential. We note that we did not include long-range electrostatic interaction via Wannier centers, as it is possible in the deepMD framework [S7], due to the much higher computational cost, only offset by a presumably small increase in accuracy.

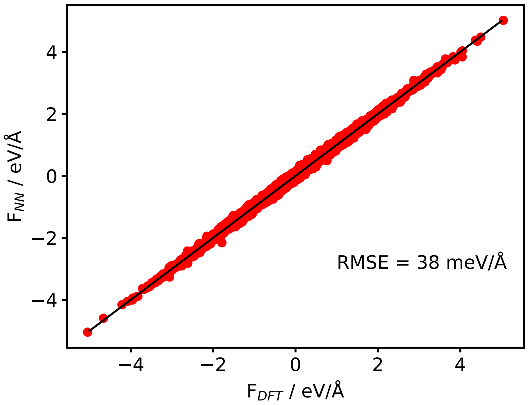

FIG. S1. Comparison of atomic forces predicted by the NNP and from DFT calculations.

* fpabst@sissa.it

In order to assess the accuracy of the NNP in predicting structural properties of water, we calculated [S8] the radial distribution function $g(r)$ of neat water and compare it to the experimental one [S9] in Fig. S2. Considering the fact that only a fraction of the training data are snapshots of neat water and the majority of the snapshots contain ions, the agreement is quite satisfactory, especially in the region of the first peak, while deviations can be seen at larger distances.

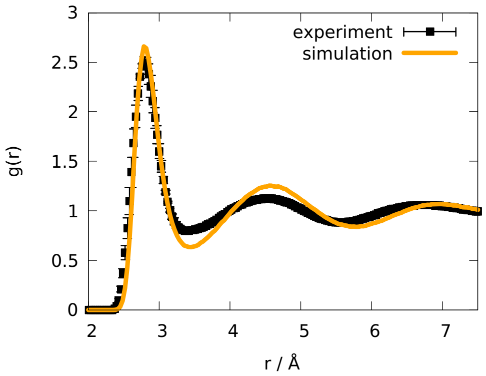

FIG. S2. Comparison of the radial distribution function as obtained with our NNP and experimental data taken from Ref. S9.

The radial distribution function of water from full ab-initio simulations using the RPBE-D3 functional are reported in Ref. S10. In comparison to the $g(r)$ obtained with the NNP shown in Fig. S2, it can be seen that our data is even in slightly better agreement with the experimental data in the region of the first peak, while the agreement is worse for the second and third peak.

II. SIMULATION DETAILS

All simulations were performed using the LAMMPS code interfaced with the DeepMD-kit [S11, S12]. The number of water molecules and ion triplets for each concentration can be seen below in table S1. Equilibration runs with a time step of 0.5 fs were performed in the NVT ensemble, where the density was set to the value obtained previously in NPT simulations. After equilibration, production runs are performed in the NVE ensemble using a time step of 0.25 fs. For each concentrations, 32 simulations are started from independent configurations of the equilibration run (separated by $\approx 10\tau$, where τ is the relaxation time) and all reported quantities are the average over these 32 runs. Equilibration and production were both run for approximately 100 times longer than the relaxation time τ.

TABLE S1. Concentrations

H_2O	ion triplets	volume (Å^3)	mol %	wt %	vol %	molarity	molality	comment
512	0	17 448	**0.00**	0.0	0.00	0.00	0.00	pure water
512	10	18 910	**1.92**	20.6	7.92	0.88	1.08	
512	20	20 446	**3.76**	34.1	14.64	1.62	2.17	
515	39	23 537	**7.04**	50.1	24.80	2.75	4.20	eutectic
513	73	29 105	**12.46**	65.4	37.55	4.16	7.90	solubility limit

III. MELTING TEMPERATURE

It is commonly observed that ab-initio simulations based on DFT, and therefore also NNP trained on DFT data, exhibit melting temperatures T_m of the liquid under study at variance with experiments [S13, S14]. The usual procedure is to "calibrate" (i.e. shift) the simulation temperature by this difference, which then commonly results in good compatibility of simulation and experiments, for instance, concerning dynamical quantities. Thus, the melting temperature for water is determined here using the interface method, i.e., the crystalline phase is brought in contact with the liquid phase and simulations are performed in the NPT ensemble at different temperatures. If the chosen temperature is below T_m, the crystalline phase is supposed to grow, and for $T > T_m$ to melt. At or very close to T_m the

two phases can coexist for quite a long time. We performed extensive simulations on 2304 molecules in the interface configuration as shown below. The lowest temperature at which we observed complete melting is $T_{\text{sim}} = 315\,\text{K}$ and the highest temperature at which we observed complete crystallization is $T_{\text{sim}} = 310\,\text{K}$. At $T_{\text{sim}} = 312.5\,\text{K}$, we observed phase coexistence for more than 25 ns. Since the experimental melting temperature of water is 273.15 K, our result is

$$T_m^{\text{sim.}} - T_m^{\text{exp.}} = 39.35\ K \pm 2.5K \tag{S1}$$

All temperatures from simulations reported in this work are shifted by this difference.

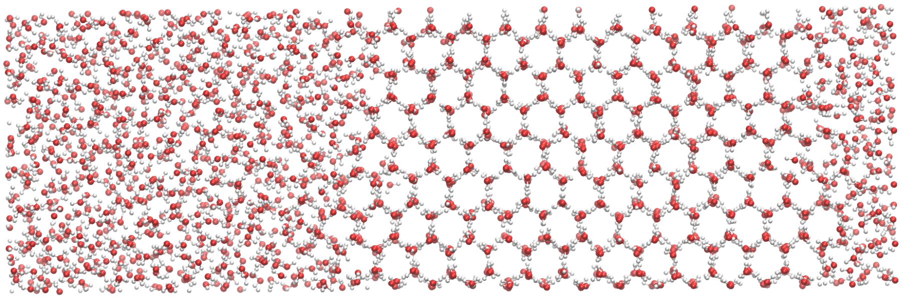

IV. CALCULATION OF THE DIELECTRIC SPECTRA

In order to calculate dielectric spectra via eq. 1 of the main manuscript, an accurate description of the dipole moment $\boldsymbol{M}$ is necessary. To this end, we use maximally localized Wannier centers [S15] to represent the position of pairs of electrons. These Wannier centers are calculated with Quantum ESPRESSO™[S2] interfaced with wannier90 [S16]. For each oxygen and each calcium ion, the four nearest Wannier centers are assigned to the respective atom. Note that in this way the ClO_4 anion gets assigned 16 Wannier centers via its four oxygens. The spatial average of four Wannier centers is called Wannier centroid (WC). A neural network, again from the DeepMD-kit [S12], is used to predict the distance vector from a WC to the respective oxygen or calcium atom. In this work, we only calculate the dielectric spectrum for H_2O molecules, see the main text. Thus, the dipole moment of a single H_2O molecule is calculated via

$$\boldsymbol{\mu}^{\text{H}_2\text{O}} = 2e \sum_{i=1}^{2} \boldsymbol{x}_i^{\text{H}} + 6e\,\boldsymbol{x}^{\text{O}} - 8e\boldsymbol{x}^{\text{WC}} \tag{S2}$$

The sum over all water dipole moments $\boldsymbol{M}^{\text{H}_2\text{O}}$ is used to calculate the total spectrum. Since the correlation function $\left\langle \boldsymbol{M}^{\text{H}_2\text{O}}(t)\boldsymbol{M}^{\text{H}_2\text{O}}(0)\right\rangle$ is still noisy even after averaging over the 32 different production runs–which is caused by the cross correlation term (see eq. 2 of the main manuscript)–we use a Savitzky-Golay filter to smooth the cross correlation term at long times before summing with the self correlation term and Fourier transforming the total correlation function. The comparison of the raw and filtered cross correlation function is shown in the left panel of figure S3. In the right panel, the total spectrum is shown with and without filtering of the cross correlation function. In this way, the raw data is influenced as little as possible, while ensuring a smooth spectrum in the frequency domain. Regarding the high frequency limiting value of $\varepsilon'(\omega)$, i.e., ε_∞ (see Eq. 2 of the main text), we used the value of 2.1 as obtained from the experimental spectrum. While the ϵ_∞ value can be easily calculated from simulations, it is notoriously difficult to get this value with very high precision from experiments using dielectric spectroscopy. In fact, in such experiments ϵ_∞ is almost always found to be larger than the expected value of $\epsilon_\infty = n^2$, with n being the refractive index. This might be due to the fact that dielectric measurements do usually not reach optical frequencies, thus overestimating ϵ_∞. However, already small differences of ϵ_∞ between experiment and simulation would render

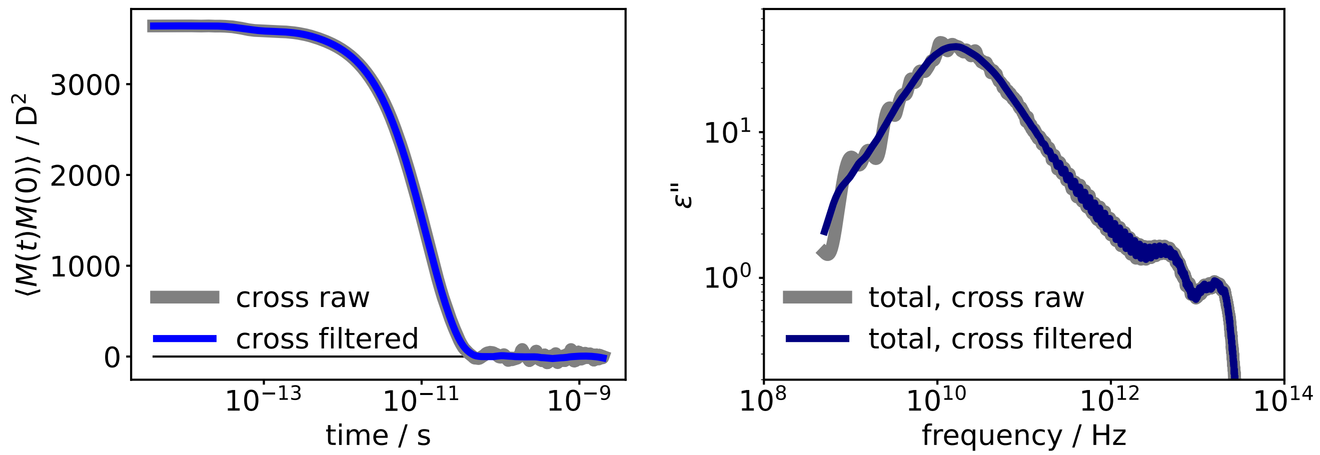

FIG. S3. Effect of filtering the long time part of the cross correlation function on the total dielectric spectrum.

the visual comparison of the ϵ' spectra in double logarithmic representation difficult at high frequencies. This is why we chose to set ϵ_∞ to the experimental value. Please note that the effect of this procedure is negligible for the value of the static dielectric constant: While the value we are using in Fig. 1 is $\epsilon_\infty = 2.1$, in Ref. [S17] it is reported to be $\epsilon_\infty = 1.88 - 1.99$ as calculated from simulations.

V. VISCOSITY

For most aqueous salt solutions an increase in viscosity with increasing salt concentration is observed. We could not find experimental values for calcium perchlorate solutions in the literature. However, we calculated the viscosity from our simulations, using the same procedure as employed in Ref. [S14]. In short, viscosity values are calculated using the sportran code [S18] for each of the 32 production runs and the final value reported in Fig. S4 is the geometric mean of these values and the error bar denotes one standard deviation. It can be seen that the viscosity increases with increasing salt concentration.

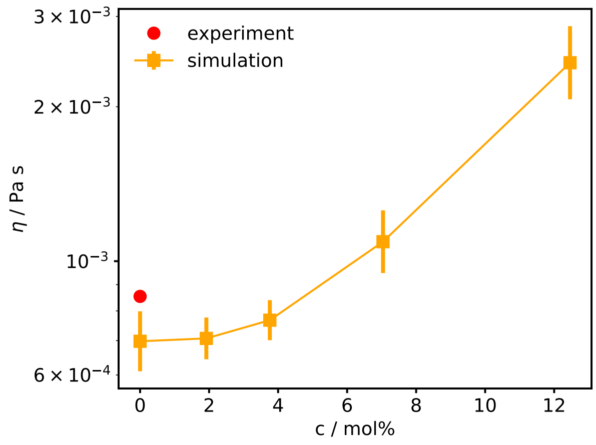

FIG. S4. Calculated viscosity values for all concentrations considered in this work. For neat water, the experimental value of the viscosity is taken from the NIST webpage [S19].

VI. HYDROGEN-BOND NETWORK

In this section we show how the structure and dynamics of the hydrogen-bond network of water changes upon adding calcium perchlorate. The reduction of the Kirkwood correlation factor as discussed in the main text is connected to a reduction in the mutual orientational correlation of dipole moments. Here, we compare this behavior to the tetrahedrality parameter q_{tet}, a common measure for the tetrahedral ordering of the hydrogen-bond network in water. It is defined as [S20]

$$q_{tet} = 1 - \frac{3}{8}\sum_{j=1}^{3}\sum_{k=j+1}^{4}\left(\cos\psi_{jk} + \frac{1}{3}\right)^2 \tag{S3}$$

where ψ_{jk} is the angle formed by the lines joining the oxygen atom of the central water molecule and its nearest neighbors i and j, and it is calculated with the order python code [S21] , taking into account all the oxygens in the simulation box, i.e., including the oxygens from the anions, which will participate in hydrogen bonding. A value of $q_{tet} = 1$ corresponds to perfect tetrahedral order, while a value of zero corresponds to its complete absence. The distribution $P(q_{tet})$ is shown in Fig. S5 and it can be seen that the peak at high q_{tet} values strongly decreases in intensity and shifts to the left, while the peak at intermediate q_{tet} values increases in intensity with increasing salt concentration. Overall, this means that the high tetrahedral order present in neat water diminishes upon addition of calcium perchlorate. This is perfectly in line with the reduced dipolar order as discussed in the main text.

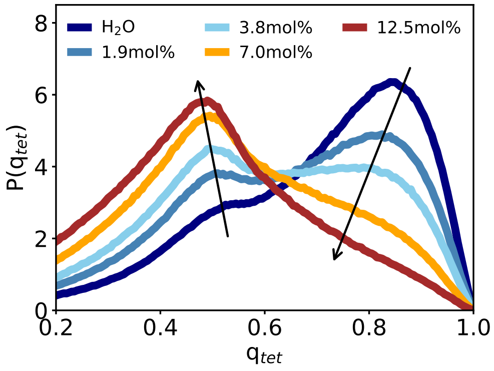

FIG. S5. Distribution of the tetrahedrality parameter q_{tet} for all salt concentrations studied.

Regarding the dynamics of the hydrogen-bond network, we consider the hydrogen-bond lifetime, calculated as follows: At time steps separated by 0.1 ps all hydrogen bonded neighbors of each water molecule are determined, using the standard definition of hydrogen bonds, i.e., a maximal O-O distance of 3.5 Å and an O-H-O angle smaller than 30° or larger than 150°, respectively. Then, the normalized autocorrelation function of the hydrogen-bonded neighbors is calculated and fitted with a stretched exponential function $\exp(-(t/\tau)^\beta)$ for the case of neat water. The correlation decays of the aqueous salt solutions exhibit an additional slow decay, which is accounted for by adding a mono-exponential decay to the fast stretched exponential decay. The correlation functions together with the fits are shown for all concentrations on the left-hand side of Fig. S6. On the right-hand side, the stretching parameter β of the fast decay is shown in the top panel and the lifetimes τ of the fast and slow decay in the lower panel. While β decreases with increasing salt concentration, indication a more heterogeneous environment of the H-bonds, the lifetimes τ are hardly affected by the salt concentration. This is true for the fast as well as the slow step. However, as can be seen on the left-hand side, the intensity of the slow decay increases with increasing salt concentration, indicating that an increasing number of H-bonds are acquiring a longer lifetime. All in all, the bimodality in the hydrogen-bond lifetime directly mirrors the observation made for the dipole reorientation time as inferred from the self part of the dielectric spectrum in the main text: For increasing salt concentrations, a bimodality in the reorientational spectrum develops, where the slower water molecules were shown to be located in the first hydration shell of the cations.

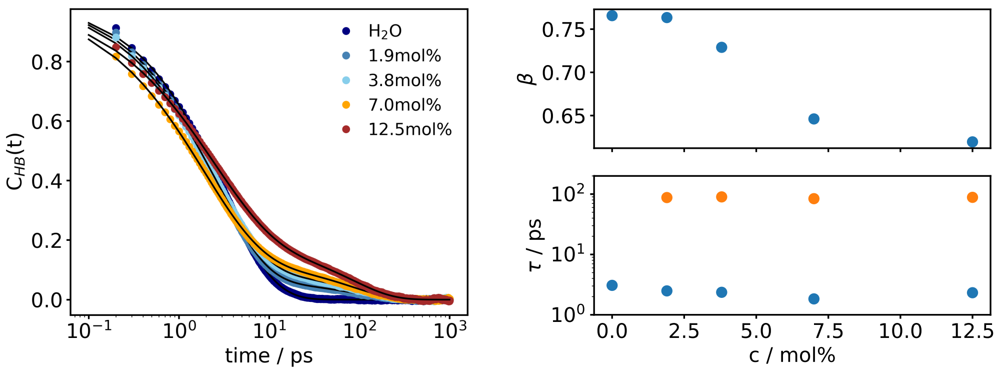

FIG. S6. Left: Correlation function of hydrogen-bonded neighbors. Black lines are fits, see text for details. Right: Stretching parameter β of the fast decay (top) and hydrogen-bond lifetimes τ as obtained for the fast and slow decay (bottom).

VII. COMPARING THE INFLUENCE OF CATION AND ANION

In the main text, we have focused on the influence of the cation on the dipolar order of the surrounding water molecules. Of course, also the anion will have an impact, but we found that the contribution of the cation is the dominating one. This is shown below in Fig. S7, where the mean angle between water dipoles are compared for different solvation shells around the cations (left panel) and anions (right panel), respectively. The top panels show the ion-oxygen radial distribution function $g(r)$ and the shaded areas denote the different solvation shells. In the lower panels the mean angle between water dipoles in the respective solvation shells are shown. It is clear that while for cations strong effects are visible already for small salt concentrations up to the third solvation shell, for anions only small effects are visible, reflecting the global trend in reduced angular correlation. This result is a direct reflection of the fact that the charge density of the cation is much higher than that of the anion and the ClO_4 anion participating in the hydrogen bonding network of water, leading to a notably smaller disruption of the dipolar order caused by the anion in comparison to the cation.

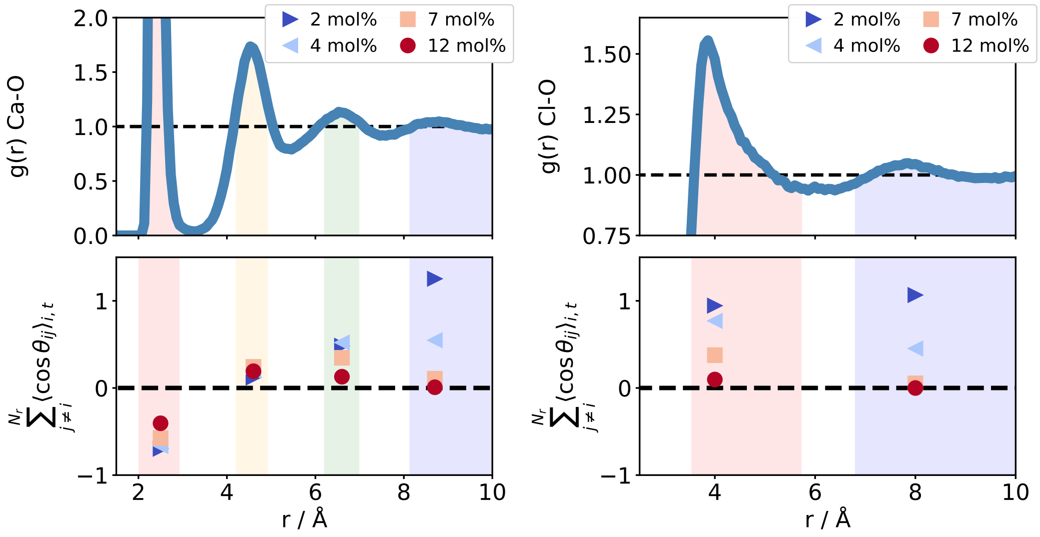

FIG. S7. Comparison of the mean angle between water dipoles in different solvation shells around the cation (left) and anion (right).

[S1] Y. Zhang, H. Wang, W. Chen, J. Zeng, L. Zhang, H. Wang, and E. Weinan, Dp-gen: A concurrent learning platform for the generation of reliable deep learning based potential energy models, Comput. Phys. Commun. **253**, 107206 (2020).

[S2] P. Giannozzi, S. Baroni, N. Bonini, M. Calandra, R. Car, C. Cavazzoni, D. Ceresoli, G. L. Chiarotti, M. Cococcioni, I. Dabo, *et al.*, Quantum espresso: a modular and open-source software project for quantum simulations of materials, J. Phys. Condens. Matter **21**, 395502 (2009).

[S3] B. Hammer, L. B. Hansen, and J. K. Nørskov, Improved adsorption energetics within density-functional theory using revised perdew-burke-ernzerhof functionals, Phys. Rev. B **59**, 7413 (1999).

[S4] S. Grimme, S. Ehrlich, and L. Goerigk, Effect of the damping function in dispersion corrected density functional theory, J. Comput. Chem. **32**, 1456 (2011).

[S5] D. Hamann, Optimized norm-conserving vanderbilt pseudopotentials, Phys. Rev. B **88**, 085117 (2013).

[S6] D. Lu, W. Jiang, Y. Chen, L. Zhang, W. Jia, H. Wang, and M. Chen, Dp compress: A model compression scheme for generating efficient deep potential models, J. Chem. Theory Comput. **18**, 5559 (2022).

[S7] L. Zhang, H. Wang, M. C. Muniz, A. Z. Panagiotopoulos, R. Car, *et al.*, A deep potential model with long-range electrostatic interactions, J. Chem. Phys. **156** (2022).

[S8] M. Brehm, M. Thomas, S. Gehrke, and B. Kirchner, Travis—a free analyzer for trajectories from molecular simulation, J. Chem. Phys **152** (2020).

[S9] A. Soper, Joint structure refinement of x-ray and neutron diffraction data on disordered materials: application to liquid water, J. Phys. Condens. Matter **19**, 335206 (2007).

[S10] K. Forster-Tonigold and A. Groß, Dispersion corrected rpbe studies of liquid water, J. Chem. Phys. **141** (2014).

[S11] A. P. Thompson, H. M. Aktulga, R. Berger, D. S. Bolintineanu, W. M. Brown, P. S. Crozier, P. J. In't Veld, A. Kohlmeyer, S. G. Moore, T. D. Nguyen, *et al.*, Lammps-a flexible simulation tool for particle-based materials modeling at the atomic, meso, and continuum scales, Comput. Phys. Commun. **271**, 108171 (2022).

[S12] J. Zeng, D. Zhang, D. Lu, P. Mo, Z. Li, Y. Chen, M. Rynik, L. Huang, Z. Li, S. Shi, *et al.*, Deepmd-kit v2: A software package for deep potential models, J. Chem. Phys. **159**, 054801 (2023).

[S13] C. Malosso, L. Zhang, R. Car, S. Baroni, and D. Tisi, Viscosity in water from first-principles and deep-neural-network simulations, Npj Comput. Mater. **8**, 139 (2022).

[S14] F. Pabst and S. Baroni, Glassy dynamics in a glass-forming liquid: A first-principles study of toluene, Phys. Rev. E **111**, L023401 (2025).

[S15] N. Marzari, A. A. Mostofi, J. R. Yates, I. Souza, and D. Vanderbilt, Maximally localized wannier functions: Theory and applications, Rev. Mod. Phys. **84**, 1419 (2012).

[S16] A. A. Mostofi, J. R. Yates, Y.-S. Lee, I. Souza, D. Vanderbilt, and N. Marzari, wannier90: A tool for obtaining maximally-localised wannier functions, Comput. Phys. Commun. **178**, 685 (2008).

[S17] C. Zhang, S. Yue, A. Z. Panagiotopoulos, M. L. Klein, and X. Wu, Why dissolving salt in water decreases its dielectric permittivity, Phys. Rev. Lett. **131**, 076801 (2023).

[S18] L. Ercole, R. Bertossa, S. Bisacchi, and S. Baroni, Sportran: A code to estimate transport coefficients from the cepstral analysis of (multivariate) current time series, Comput. Phys. Commun. **280**, 108470 (2022).

[S19] National Institute of Standards and Technology, `https://webbook.nist.gov/chemistry/fluid/`.

[S20] J. R. Errington and P. G. Debenedetti, Relationship between structural order and the anomalies of liquid water, Nature **409**, 318 (2001).

[S21] `https://github.com/ipudu/order`.
