# Supplementary material for: How Salt Solvation Slows Water Dynamics While Blue-Shifting Its Dielectric Spectrum
Source: J Phys Chem Lett. 2025 Jul 28;16(31):7915–20. doi: 10.1021/acs.jpclett.5c01401 (PMC12337140; doi:10.1021/acs.jpclett.5c01401)
Supplement: Supplementary file 2 [file jz5c01401_si_002.pdf]

jz-2025-01401t.R1

Name: Peer Review Information for "How Salt Solvation Slows Water Dynamics While Blue-Shifting Its Dielectric Spectrum"

First Round of Reviewer Comments

Reviewer: 1

Comments to the Author

In this work, the authors study the dielectric losses of saline water. By using correlation functions and calculating the dielectric spectra, they demonstrate a connection between the decrease in the dielectric constant and the orientational ordering of water molecules. This topic has already been explored by X. Wu and co-workers, who showed that the dielectric loss is primarily driven by the disruption of intermolecular correlations due to the intrusion of ionic hydration shells into the hydrogen-bond network.

In the present study, the authors adopt a similar methodological framework but apply it to perchlorate ions, introducing some originality compared to the earlier work. Furthermore, like X. Wu, they use a machine learning-based force field to describe interactions at the ab initio level, which enables them to account for the polarizability of both ions and water molecules. Their main finding is that the reduction in the dielectric constant stems from the degradation of orientational cross-correlations among water molecules in the immediate vicinity of a solvated cation — a result consistent with the insights of Wu et al.

Although the paper is well written, the narrative is coherent, and the main result appears significant, I believe that additional discussion and calculations are necessary.

In particular, no microscopic insights supporting the calculated cross-correlations are provided. A detailed microscopic picture is needed to capture the evolution of the hydrogen-bonding network — such as the tetrahedral order parameter, hydrogen bond

lifetimes, residence times, and excluded volume effects. Moreover, recent studies have focused on the reduction of the dielectric constant in nanoconfined water (e.g., Baggi et al. and Laage et al.). The authors should compare and contrast their interpretations and the underlying mechanisms with those proposed by Baggi and Laage and co-workers. Can the two explanations be reconciled or connected?

To strengthen the impact of the paper and clearly distinguish it from the work of X. Wu, the authors should include a more in-depth discussion on how their conclusions relate to the expected effects of confinement on the dielectric constant.

Reviewer: 2

Comments to the Author

The manuscript, titled “How Salt Solvation Slows Water Dynamics While Blue-Shifting Its Dielectric Spectrum,” presents a first-principles study on the intriguing phenomenon in salt solutions where the imaginary part of the dielectric response exhibits a blue shift with increasing concentration, while viscosity decreases, a seemingly counterintuitive effect. The authors successfully explain this phenomenon by attributing the blue shift primarily to cross-correlation effects within the solvation shell, particularly at higher concentrations, while the self-correlation contribution undergoes an expected red shift. This work provides a significant contribution to chemical physics by clarifying these effects through physical principles. It is suitable for publication in The Journal of Physical Chemistry Letters (JPCL), pending the authors’ response to the following concerns and questions, including some critical points about the analyses and methodology:

1. Anticorrelation Explanation in Figure 3: The manuscript attributes the anticorrelation observed in the inset of Figure 3 solely to water molecules in the first solvation shell of the cation (Ca^{2+}). While this explanation is partially correct, it overlooks the symmetry-breaking effect, where the tetrahedral water structure in bulk water transitions to a spherical arrangement around ions. This effect, also applicable to the anion (ClO_4^-), likely contributes to the observed anticorrelation, as similarly noted for Cl^- in Figure 3 of Ref. 18. The authors should address the anion’s role in this anticorrelation.

2. Relative Impact of Cations vs. Anions: Related to point 1, the manuscript does not clarify whether the cation (Ca^{2+}) or anion (ClO_4^-) has a greater influence on the blue shift of the imaginary part of the dielectric response. A discussion comparing their contributions would strengthen the analysis.

3. Viscosity Discussion: The title and abstract highlight viscosity as a key property, yet it is neither discussed nor presented in the manuscript. I strongly recommend including theoretical predictions of viscosity, either in the main text or supplementary materials, to validate the model's ability to reproduce experimental viscosity trends qualitatively.

4. High-Frequency Dielectric Constant ($\varepsilon\infty$): The authors use experimental values for $\varepsilon\infty$ instead of computing them. Given that $\varepsilon\infty$ is relatively straightforward to calculate and converges quickly over disordered configurations in solutions (as shown in Ref. 18), the authors should justify this choice.

5. Structural Comparisons: The manuscript demonstrates excellent agreement between experimental and theoretical real and imaginary parts of the dielectric function. However, it lacks comparisons of structural properties, such as pair distribution functions or structural factors from neutron scattering experiments. Additionally, viscosity comparisons are absent. Including these would enhance the study's robustness.

6. Dipolar Correlation Decomposition: Equation (3) decomposes the overall dipolar correlation into self-correlation and cross-correlation components. This approach, while effective, is not novel, as it was first introduced by Roberto Car's group in M. Sharma, R. Resta, and R. Car, Phys. Rev. Lett. 98, 247401 (2007). The authors should acknowledge this prior work.

7. Neural Network Training for Dipole Moments: The manuscript notes that the neural network was trained using DeepMD. However, it is unclear how the dipole moments of water molecules were trained. Were Wannier centers used and trained separately? If so, relevant references for this methodology should be cited.

8. Long-Range Coulombic Forces in DeepMD: It is unclear whether long-range Coulombic interactions are included in the DeepMD training. While these interactions may not be critical, a brief discussion of their inclusion or exclusion would be valuable for readers.

9. Linear Relationship at $8\tau_0$: The final paragraph states that "longer than $8\tau_0$, ... a linear relationship ... is observed." The authors should provide a qualitative discussion of the significance of the $8\tau_0$ threshold to clarify its physical relevance.

Addressing these points will strengthen the manuscript and support a positive recommendation for publication.

Author's Response to Peer Review Comments:

We would like to thank both referees for their positive assessment of our work and the constructive feedback which helped us to notably improve our manuscript. Please find our answers to the questions raised by the referees below, together with the indication of changes made to the manuscript, which are highlighted in red.

Reviewer: 1

Recommendation: This paper may be publishable, but major revision is needed; I would like to be invited to review any future revision.

Comments: In this work, the authors study the dielectric losses of saline water. By using correlation functions and calculating the dielectric spectra, they demonstrate a connection between the decrease in the dielectric constant and the orientational ordering of water molecules. This topic has already been explored by X. Wu and co-workers, who showed that the dielectric loss is primarily driven by the disruption of intermolecular correlations due to the intrusion of ionic hydration shells into the hydrogenbond network.

In the present study, the authors adopt a similar methodological framework but apply it to perchlorate ions, introducing some originality compared to the earlier work. Furthermore, like X. Wu, they use a machine learning-based force field to describe interactions at the ab initio level, which enables them to account for the polarizability of both ions and water molecules. Their main finding is that the reduction in the dielectric constant stems from the degradation of orientational cross-correlations among water molecules in the immediate vicinity of a solvated cation — a result consistent with the insights of Wu et al.

Answer: We would like to point out that while our results for the dielectric constant are in full agreement with the one by Wu and coworkers (for a distinctly different salt), a fact which we explicitly state in our manuscript, our main focus is not the reduction in the dielectric

constant. Instead, we go beyond the static regime explored by Wu and coworkers and focus on the dynamics, i.e., the frequency dependent dielectric spectrum. In our opinion this fact is clearly stated throughout our manuscript, starting with the title.

Although the paper is well written, the narrative is coherent, and the main result appears significant, I believe that additional discussion and calculations are necessary.

In particular, no microscopic insights supporting the calculated cross-correlations are provided. A detailed microscopic picture is needed to capture the evolution of the hydrogen-bonding network — such as the tetrahedral order parameter, hydrogen bond lifetimes, residence times, and excluded volume effects.

Answer: **We agree with the referee that microscopic insights in the hydrogen-bond network, and its disruption by the ions, will help the reader to get a better picture of the meaning of the dipolar cross-correlations. We note that the residence time, as requested by the referee, is already shown in Fig. 5 of the manuscript and discussed in the text. We therefore studied the tetrahedral order parameter as well as the hydrogen bond lifetime, as suggested by the referee. We find that the tetrahedrality is notably reduced with increasing salt concentrations, reflecting the fact that the highly ordered hydrogen-bond network present in water is disrupted by the presence of the ions, leading to a reduction in orientational cross-correlations. The mean hydrogen-bond lifetime stays approximately constant for all concentrations, however, the distribution of the lifetimes notably broadens when adding salt and an additional long-time decay develops. The former reflects the more heterogeneous environment with added ions, while the latter directly reflects the presence of slowly reorienting water molecules, as discussed in the manuscript. In the revised manuscript, the outcome of this hydrogen-bond analysis is shown in section VI of the supporting information and we added a summary of it in two places in the main text, one discussing the structural aspect, and the other one the dynamic findings.**

Moreover, recent studies have focused on the reduction of the dielectric constant in nanoconfined water (e.g., Baggi et al. and Laage et al.). The authors should compare and contrast their interpretations and the underlying mechanisms with those proposed by Baggi and Laage and co-workers. Can the two explanations be reconciled or connected?

Answer: **We agree with the referee that water in confinement is topic of high interest. However, we could not find any study on this subject by an author with the name Baggi. We assume that the referee meant Bagchi instead. Therefore, we included in the revised paper a paragraph summarizing the findings of Laage et al. and Bagchi et al. for water in confinement and compare it to our findings.**

To strengthen the impact of the paper and clearly distinguish it from the work of X. Wu, the authors should include a more in-depth discussion on how their conclusions relate to the expected effects of confinement on the dielectric constant.

Answer: As mentioned above, we believe that our paper is clearly distinguished from the work of Wu and coworkers by the fact that we focus on the dynamics instead of the static dielectric constant. Nevertheless, we acknowledge that the effect of confinement on the dielectric constant is a hot topic and—as mentioned above—we therefore included in the revised manuscript a paragraph summarizing the findings and containing a discussion about its connection to our work.

Urgency: High

Significance: High

Novelty: High

Scholarly Presentation: High

Is the paper likely to interest a substantial number of physical chemists, not just specialists working in the authors' area of research?: Yes

---

Reviewer: 2

Recommendation: This paper may be publishable, but major revision is needed; I would like to be invited to review any future revision.

Comments: The manuscript, titled "How Salt Solvation Slows Water Dynamics While Blue-Shifting Its Dielectric Spectrum," presents a first-principles study on the intriguing phenomenon in salt solutions where the imaginary part of the dielectric response exhibits a blue shift with increasing concentration, while viscosity decreases, a seemingly counterintuitive effect. The authors successfully explain this phenomenon by attributing the blue shift primarily to cross-correlation effects within the solvation shell, particularly at higher concentrations, while the self-correlation contribution undergoes an expected red shift. This work provides a significant contribution to chemical physics by clarifying these effects through physical principles. It is suitable for publication in The Journal of Physical Chemistry Letters (JPCL), pending the authors' response to the following concerns and questions, including some critical points about the analyses and methodology:

1. Anticorrelation Explanation in Figure 3: The manuscript attributes the anticorrelation observed in theinset of Figure 3 solely to water molecules in the first solvation shell of the cation (Ca^{2+}). While this explanation is partially correct, it overlooks the symmetry-breaking effect, where the tetrahedral water structure in bulk water transitions to a spherical arrangement around ions. This effect, also applicable to the anion (ClO^{-}_{4}), likely contributes to the observed anticorrelation, as similarly noted for Cl^{-} in Figure 3 of Ref. 18. The authors should address the anion's role in this anticorrelation.

Answer: **The referee is completely right in remarking that also the anion will have an influence on the ordering of the water molecules. In fact, we checked that the influence of the cation is much larger, and thus focused on the cation. However, we missed to include a statement about this fact in our manuscript. In the revised version, we added a sentence to the main text and a section to the supporting information discussing the influence of the anion.**

2. Relative Impact of Cations vs. Anions: Related to point 1, the manuscript does not clarify whetherthe cation (Ca^{2+}) or anion (ClO^{-}_{4}) has a greater influence on the blue shift of the imaginary part of the dielectric response. A discussion comparing their contributions would strengthen the analysis.

Answer: **As mentioned above in our response to the first point, we find that the influence of the cation is much stronger, which is why we focused on the cation. Nevertheless, the role of the anion needs to be discussed and we do so in the revised manuscript. We added a**

sentence to the main text and a section to the supporting information discussing the influence of the anion.

3. Viscosity Discussion: The title and abstract highlight viscosity as a key property, yet it is neither discussed nor presented in the manuscript. I strongly recommend including theoretical predictions of viscosity, either in the main text or supplementary materials, to validate the model's ability to reproduce experimental viscosity trends qualitatively.

Answer: We completely agree with the referee. We now calculated the viscosity for all concentrations studied. It can be seen that the viscosity increases with increasing salt concentration. We added a sentence about these calculations to the main text and present the viscosities in section V of the supporting information.

4. High-Frequency Dielectric Constant (ϵ_∞): The authors use experimental values for ϵ_∞ instead of computing them. Given that ϵ_∞ is relatively straightforward to calculate and converges quickly over disordered configurations in solutions (as shown in Ref. 18), the authors should justify this choice.

Answer: While it is true that the ϵ_∞ value is easily calculated, it is notoriously difficult to get this value with very high precision from experiments using dielectric spectroscopy. In fact, in such experiments ϵ_∞ is almost always found to be larger than the expected value of $\epsilon_\infty = n^2$, with n being the refractive index. This might be due to the fact that dielectric measurements do usually not reach optical frequencies, thus overestimating ϵ_∞. However, already small differences of ϵ_∞ between experiment and simulation would render the visual comparison of the ϵ' spectra in double logarithmic representation difficult at high frequencies. This is why we chose to set ϵ_∞ to the experimental value. Please note that the effect of this procedure is negligible for the value of the static dielectric constant: While the value we are using in Fig. 1 is $\epsilon_\infty = 2.1$, in Ref. 18 (Ref. 19 of the revised manuscript) it is reported to be $\epsilon_\infty = 1.88 - 1.99$ as calculated from simulations. In the revised manuscript, we added this discussion to section IV of the supporting information.

5. Structural Comparisons: The manuscript demonstrates excellent agreement between experimental and theoretical real and imaginary parts of the dielectric function. However, it lacks comparisons of structural properties, such as pair distribution functions or structural factors from neutron scattering experiments. Additionally, viscosity comparisons are absent. Including these would enhance the study's robustness.

Answer: We agree with the referee that structural quantities are usually used as a first measure to assess the agreement between simulations and experiments. Therefore, we include in the SI the comparison of the simulated pair distribution function with the experimental one. It can be seen that the agreement at the first peak is even better than

reported for ab-initio simulations of pure water using the RPBE functional with D3 dispersion correction in J. Chem. Phys. 141, 064501 (2014). However, the agreement is slightly worse at the second and third peak.

As mentioned above, we included viscosity data from the simulations to the supporting information of the revised manuscript and also compare it to the experimental value of neat water (there is no experimental data available on calcium perchlorate solutions).

6. Dipolar Correlation Decomposition: Equation (3) decomposes the overall dipolar correlation into self-correlation and cross-correlation components. This approach, while effective, is not novel, as it was first introduced by Roberto Car's group in M. Sharma, R. Resta, and R. Car, Phys. Rev. Lett. 98, 247401 (2007). The authors should acknowledge this prior work.

Answer: We completely agree that this decomposition is not new. In fact, it can be considered textbook knowledge, employed long before the work mentioned by the referee. However, the mentioned reference is certainly important in the present context, and we therefore cite it in the revised manuscript below Eq. 1, while citing a textbook when introducing Eq. 3.

7. Neural Network Training for Dipole Moments: The manuscript notes that the neural network was trained using DeepMD. However, it is unclear how the dipole moments of water molecules were trained. Were Wannier centers used and trained separately? If so, relevant references for this methodology should be cited.

Answer: The training of the dipole model is explained in detail in section IV of the supporting information. Wannier centers are used for this task and the relevant references are cited. In the main text of the revised manuscript we now include a sentence about that after we explain the training of the NNP.

8. Long-Range Coulombic Forces in DeepMD: It is unclear whether long-range Coulombic interactionsare included in the DeepMD training. While these interactions may not be critical, a brief discussion of their inclusion or exclusion would be valuable for readers.

Answer: Thanks for pointing this out. Indeed, long-range Coulombic interactions are only implicitly included. The explicit inclusion, which is also possible in the deepMD framework, is not employed mainly due to the much higher computational costs, which are only offset by a small presumed improvement in accuracy. In the revised manuscript we include this statement in the supporting information.

9. Linear Relationship at $8\tau_0$: The final paragraph states that "longer than $8\tau_0$, … a linear relationship … is observed." The authors should provide a qualitative discussion of the significance of the $8\tau_0$ threshold to clarify its physical relevance.

<u>Answer:</u> At the moment we can not give any insights into this factor of 8. To explore the physical relevance of this factor, we are now working with different salts, which will be discussed in a forthcoming publication. Here, we can only state that the precise value of this factor likely depends on many subtleties like the charge density of the ions, the number of water molecules in the solvation shell, etc. In the revised manuscript we added a sentence stating that the origin of the factor of 8 has to be left open for further research.

Addressing these points will strengthen the manuscript and support a positive recommendation for publication.

Urgency: High

Significance: Top 10%

Novelty: High

Scholarly Presentation: High

jz-2025-01401t.R2

Name: Peer Review Information for "How Salt Solvation Slows Water Dynamics While Blue-Shifting Its Dielectric Spectrum"

Second Round of Reviewer Comments

Reviewer: 1

Comments to the Author

The authors have satisfactorily answered my questions; the article is therefore suitable for publication

Author's Response to Peer Review Comments:

1. Done
2. Done
3. The reference number refers only to data. The graphic was NOT published previously.
4. Done
5. Done
6. Done
